# In Vitro and In Vivo Efficacies of the Linear and the Cyclic Version of an All-d-Enantiomeric Peptide Developed for the Treatment of Alzheimer’s Disease

**DOI:** 10.3390/ijms22126553

**Published:** 2021-06-18

**Authors:** Sarah Schemmert, Luana Cristina Camargo, Dominik Honold, Ian Gering, Janine Kutzsche, Antje Willuweit, Dieter Willbold

**Affiliations:** 1 Institute of Biological Information Processing, Structural Biochemistry (IBI-7), Forschungszentrum Jülich, 52425 Jülich, Germany; s.schemmert@fz-juelich.de (S.S.); l.camargo@fz-juelich.de (L.C.C.); d.honold@fz-juelich.de (D.H.); i.gering@fz-juelich.de (I.G.); j.kutzsche@fz-juelich.de (J.K.); 2 Institut für Physikalische Biologie, Heinrich-Heine-Universität Düsseldorf, 40225 Düsseldorf, Germany; 3 Institute of Neuroscience and Medicine, Medical Imaging Physics (INM-4), Forschungszentrum Jülich, 52425 Jülich, Germany; a.willuweit@fz-juelich.de

**Keywords:** Alzheimer’s disease, d-peptides, treatment, behavior, Tg-SwDI mice, cognition, disassembly, Aβ, oligomers

## Abstract

Multiple sources of evidence suggest that soluble amyloid β (Aβ)-oligomers are responsible for the development and progression of Alzheimer’s disease (AD). In order to specifically eliminate these toxic Aβ-oligomers, our group has developed a variety of all-d-peptides over the past years. One of them, RD2, has been intensively studied and showed such convincing in vitro and in vivo properties that it is currently in clinical trials. In order to further optimize the compounds and to elucidate the characteristics of therapeutic d-peptides, several rational drug design approaches have been performed. Two of these d-peptides are the linear tandem (head-to-tail) d-peptide RD2D3 and its cyclized form cRD2D3. Tandemization and cyclization should result in an increased in vitro potency and increase pharmacokinetic properties, especially crossing the blood–brain-barrier. In comparison, cRD2D3 showed a superior pharmacokinetic profile to RD2D3. This fact suggests that higher efficacy can be achieved in vivo at equally administered concentrations. To prove this hypothesis, we first established the in vitro profile of both d-peptides here. Subsequently, we performed an intraperitoneal treatment study. This study failed to provide evidence that cRD2D3 is superior to RD2D3 in vivo as in some tests cRD2D3 failed to show equal or higher efficacy.

## 1. Introduction

Our society is aging and, with it, the number of diseases of old age, especially dementias, are increasing [1]. Alzheimer’s disease (AD) is a devastating neurodegenerative disorder and the most common form of dementia worldwide. Its clinical symptoms are considered to be disturbances of memory, language, spatial and temporal orientation and cognitive decline. The pathological hallmarks of the disease are characterized by neurodegeneration, intracellular depositions of neurofibrillary tangles and extracellular accumulations of amyloid-β (Aβ) plaques. Aβ is the product of the proteolytic processing of the amyloid precursor protein (APP), which is cleaved by β- and γ-secretases, resulting in Aβ-monomers. Due to unknown reasons, these monomers aggregate into Aβ-oligomers and insoluble Aβ-fibrils, which compact further into senile Aβ-plaques [2,3]. For a long time, it was assumed that Aβ-plaques were responsible for the disease and the cognitive decline of affected patients. Currently, however, the aforementioned soluble Aβ-oligomers are regarded as more than merely an intermediate on their way from monomers to plaques. Instead, they are postulated to be the most toxic species, responsible for synapse deterioration, neuronal death, disrupted Ca^2+^-homeostasis and dysfunctional plasticity—all in all leading to the clinical symptoms of AD [4]. In addition, there is increasing evidence that Aβ-oligomers are able to replicate in a prion-like fashion [5]. In our group, we are focused on the development of compounds for a disease modifying or even curative treatment of AD. For this purpose, we designed all-d-enantiomeric peptides to directly destabilize, disassemble and ultimately eliminate toxic Aβ-oligomers via direct disruption, rather than relying on the immune system for their degradation. By use of mirror image phage display against monomeric Aβ, the lead compound D3, a d-enantiomeric peptide consisting of 12 amino acid residues, arose [6]. d-peptides have several advantages as therapeutics compared to their l-enantiomeric equivalents. As shown by our group and others, the proteolytic stability of d-peptides is superior to l-peptides with the same sequence of amino acid residues, because proteases are most often stereoselective for l-amino acid residues [7]. This results also in a reduced immunogenicity and increased bioavailability of d-peptides [8]. In order to optimize the lead structure, with respect to increased stability, affinity to Aβ(1-42), blood–brain-barrier (BBB) penetration, and in vitro potency and in vivo efficacy, rational drug design approaches were conducted. The most promising compound so far, called RD2, is currently under development for the treatment of patients with AD [9]. The suggested mode of action was demonstrated by successful target engagement in vitro and in vivo [10,11]. Furthermore, the in vivo efficacy of RD2 was proven in different AD mouse models, even in old-aged mice with fully developed AD-associated pathology [10,11,12,13]. Besides RD2, more d-peptides were developed out of a rational drug design. In order to combine the favorable properties of D3 and RD2, a heteromeric linear head-to-tail version of both d-peptides, called RD2D3, was designed. Compared to RD2 and D3, we have shown increased binding affinity to Aβ(1-42) [14] and a higher potency to eliminate toxic Aβ-oligomers for RD2D3 [12]. In vivo, it was demonstrated that RD2D3 has a favorable pharmacokinetic profile (after intraperitoneal (i.p.) administration), high proteolytic stability and therapeutic efficacy by improving the cognitive abilities of transgenic APP Swedish/Dutch/Iowa (Tg-SwDI) AD mice [14,15]. Besides the development of linear tandem-d-peptides, a further approach for a rational drug design was the cyclization of either homo- or tandem-d-peptides [16,17]. For the cyclized tandem-d-peptide cRD2D3, a remarkably enhanced pharmacokinetic profile was found [17]. Comparing the pharmacokinetic profiles of the linear and cyclic version of the d-peptide RD2D3 (RD2D3 vs. cRD2D3) after intravenous (i.v.). and intraperitoneal (i.p.) administration in C57Bl/6 wild type (WT) mice resulted in a tremendously increased terminal half-life (2.3 vs. 58 h), increased BBB penetration values and brain peptide concentrations of cRD2D3. Furthermore, for cRD2D3 a high oral bioavailability was demonstrated [17]. Both, a long half-life and high oral bioavailability are extremely advantageous for active ingredients used in the therapy of AD and qualify for daily drug administrations. Since it can be assumed that AD therapy will take place over a longer period of time (possibly several years to decades), a therapy regime with once-daily oral drug intake is most convenient for the patient (treatment adherence).

Here, we wanted to investigate whether the more favorable pharmacokinetic profile of the cyclic d-peptide cRD2D3 compared to its linear version RD2D3 results in an increased in vivo efficacy. Before investigating the in vivo efficacy of RD2D3 and cRD2D3 in the Tg-SwDI AD mouse model, we performed an in-depth in vitro characterization of the compounds on the specific Aβ-mutation, which was introduced to the Tg-SwDI AD mouse model in comparison to wild type Aβ(1-42). This Aβ mutation is the so called “Dutch” and “Iowa” mutation (D/I Aβ) and it is described by an exchange of the amino acid glutamate (E) for glutamine (Q) at position 22 and of aspartate (D) for asparagine (N) at position 23 of the Aβ(1-42) sequence.

## 2. Results

### 2.1. The Cyclic d-Peptide cRD2D3 Revealed Increased In Vitro Potency Compared to the Linear d-Peptide RD2D3 Without Differences in the Proteolytic Stability

**Figure 1:**
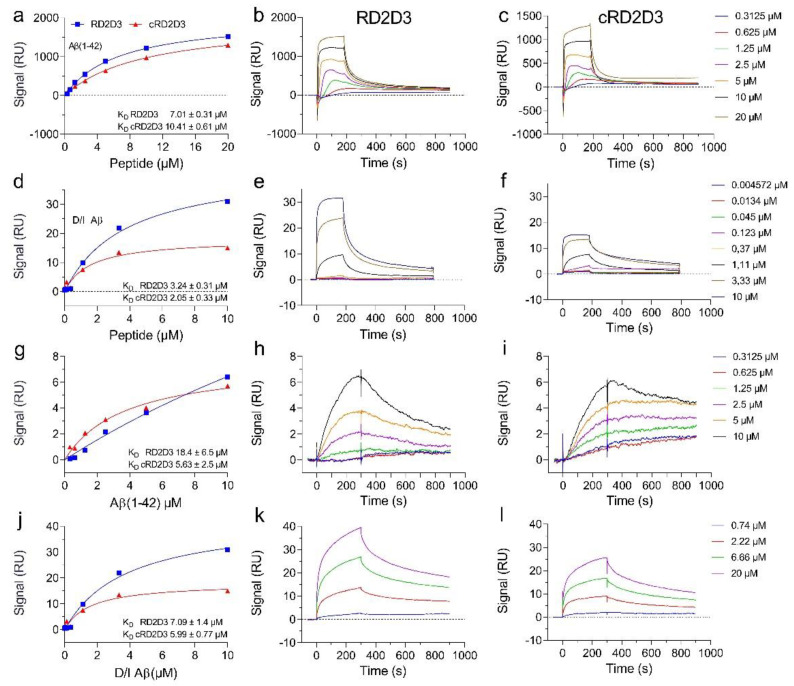
Affinity determination of RD2D3 and cRD2D3 to Aβ(1-42) and D/I Aβ by SPR. By use of the d-peptides as analyte, either Aβ(1-42) or D/I Aβ was immobilized on a CM-5 sensor chip and the binding of different RD2D3 (blue) and cRD2D3 (red) concentrations were analyzed in a multi cycle experiment (**a**–**f**). Real-time surface plasmon resonance (SPR) sensorgrams of different RD2D3 and cRD2D3 concentrations (left). Equilibrium dissociation constants (K_D_) were determined by using a Langmuir 1:1 binding model (RD2D3 middle, cRD2D3 right). RD2D3 or cRD2D3 were immobilized on a series S CM-5 sensor chip, and the binding of Aβ(1-42) or D/I Aβ monomers as an analyte at various concentrations was observed (**g**–**l**). For evaluation, the steady-state binding signals were plotted over the concentrations and fitted using a Langmuir 1:1 binding model (**a**,**d**,**g**,**j**).

In order to investigate the in vitro potency of the cyclic d-peptide cRD2D3 and its linear form RD2D3, we performed several experiments concerning the binding of the d-peptides to Aβ(1-42) and D/I Aβ. By use of surface plasmon resonance (SPR) measurements, we determined the dissociation constant (K_D_) of RD2D3 and cRD2D3 to both Aβ-species, by using Aβ as ligand and the d-peptide as analyte or vice versa. As illustrated in Figure 1, both d-peptides bound with µM concentrations to Aβ(1-42) and to D/I Aβ. By use of Aβ(1-42) or D/I Aβ as an analyte, both d-peptides bound with higher affinity to D/I Aβ (K_D_ Aβ(1-42): RD2D3 7.01 ± 0.31 µM, cRD2D3 10.41 ± 0.61 µM; D/I Aβ: RD2D3 3.24 ± 0.31 µM, cRD2D3 2.05 ± 0.33, Figure 1). By use of the d-peptides as a ligand, cRD2D3 bound with an almost identical affinity to both Aβ species (K_D_ Aβ(1-42): 5.63 ± 2.5 µM, K_D_ D/I Aβ 5.99 ± 0.77 µM, Figure 1). For RD2D3, the picture is different. The d-peptide bound with half the affinity to Aβ(1-42) than to D/I Aβ (K_D_ Aβ(1-42): 18.4 ± 6.5 µM, K_D_ D/I Aβ 7.09 ± 1.4 µM, Figure 1).

**Figure 2:**
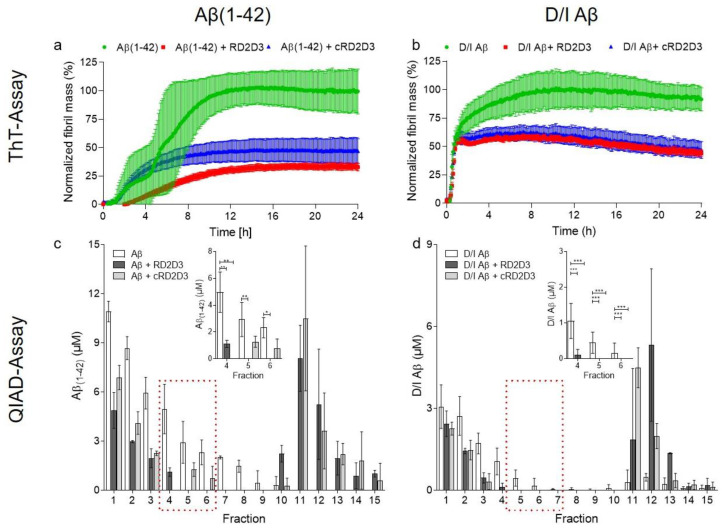
RD2D3 and cRD2D3 inhibited the Aβ(1-42) and D/I Aβ fibril formation and eliminated significantly toxic oligomers. ThT-Assay (upper panel). Both d-peptides, RD2D3 (red) and cRD2D3 (blue), were analyzed according to their ability to inhibit either the Aβ(1-42) (**a**,**left**) or the D/I Aβ (**b**,**right**) fibril formation by use of equimolar concentrations (10 µM). All analyzed d-peptides were able to inhibit either the Aβ(1-42) (**a**) or the D/I Aβ (both green) (**b**) fibril formation. Fibril mass was normalized to the Aβ control. Data are presented as mean ± SD (*N* = 3 out of three independent experiments). QIAD-Assay (lower panel). Aβ(1-42) (**c**,**left**) and D/I Aβ (**d**,**right**) size distribution without (white) or with d-peptide were analyzed by density gradient centrifugation with subsequent measurements of the Aβ concentrations. Aβ-oligomers are located in fractions 4–6. Comparison of 20 µM RD2D3 (dark grey) and 20 µM cRD2D3 (light grey) (**c**) revealed similar Aβ(1-42) (80 µM) oligomer elimination efficacy of both d-peptides. Comparison of 10 µM RD2D3 (dark grey) and 10 µM cRD2D3 (light grey) (**d**) revealed higher potency of RD2D3 to eliminate toxic D/I Aβ (40 µM)-oligomers than cRD2D3 does. Data are presented as mean ± SD (*N* = 2–5) *** *p* < 0.001, ** *p* < 0.01 and * *p* < 0.05.

The ability to inhibit the fibril formation of Aβ is of great importance for substances developed for a treatment of AD. In order to test for interference with fibril formation, we performed a thioflavin T test (ThT). The fluorescence signal of ThT, a benzothiazole dye, increases upon binding to amyloid fibrils. In order to investigate the functional effects of RD2D3 and cRD2D3 on Aβ(1-42) and on D/I Aβ, the d-peptides were incubated with either Aβ(1-42) or D/I Aβ (equimolar concentrations) and 5 µM ThT. ThT fluorescence was monitored every 6 min for 24 h. As demonstrated in Figure 2a,b, both d-peptides were able to inhibit the Aβ(1-42) and D/I Aβ fibril formation. Interestingly, this effect was more pronounced on Aβ(1-42) than on D/I Aβ (Figure 2a,b). Besides the potency of RD2D3 and cRD2D3 to inhibit the Aβ fibril formation, we also investigated the potency of both compounds to eliminate toxic Aβ-oligomers. For this, the so called QIAD (quantitative determination of interference with Aβ aggregate size distribution) assay [18] was conducted with both d-peptides and Aβ(1-42) and D/I Aβ. The outcome of the QIAD assay revealed that both RD2D3 and cRD2D3 were capable to significantly eliminate toxic Aβ(1-42)-oligomers (RD2D3 89%, cRD2D3 80%, Figure 2c, one-way ANOVA, with Bonferroni post hoc analysis, Aβ(1-42) vs. RD2D3 fraction 4 *p* = 0.001, fraction 5, *p* = 0.011, fraction 6 *p* = 0.01, cRD2D3 fraction 4 *p* = 0.005, fraction 5 *p* = 0.12, fraction 6 *p* = n.s. (0.057)). Furthermore, both d-peptides eliminated toxic D/I Aβ-oligomers with a similar potency (RD2D3 94%, cRD2D3 100%, Figure 2d, not significant for cRD2D3 despite the total elimination of toxic oligomers, one-way ANOVA with Bonferroni post hoc analysis, D/I Aβ vs. RD2D3 fraction 4 *p* = n.s.(0.062), fraction 5, *p* = 0.011, fraction 6 *p* = n.s, cRD2D3 fraction 4 *p* = n.s. (0.094), fraction 5 *p* = (n.s.) 0.118 and fraction 6 *p* = n.s.).

**Figure 3:**
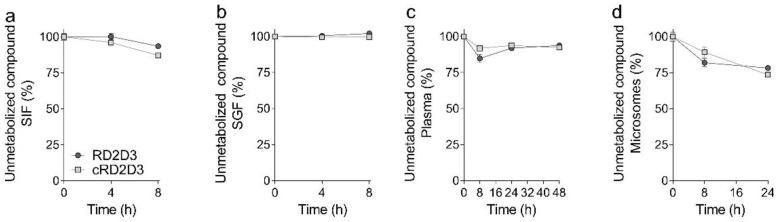
Proteolytic stability of RD2D3 and cRD2D3 in different human (simulated) body fluids. Both d-peptides, RD2D3 and cRD2D3, are remarkably stable in simulated intestinal and gastric fluid (SIF and SGF) (**a**,**b**), and in human plasma (**c**) and human liver microsomes (**d**). Shown are data form three independent experiments (mean ± SD).

In order to verify and extend the proteolytic stability/in vitro ADME (absorption, distribution, metabolism and excretion) of RD2D3 and cRD2D3, previously described for tritium labeled d-peptides (^3^H) [15,17], we performed different tests to investigate the proteolytic stability in different (simulated) body fluids. In both, simulated gastric (SGF) and intestinal fluid (SIF), RD2D3 and cRD2D3 were remarkably stable (RD2D3: SIF 4 h 100%, 8 h 93.5%, SGF 4 h 100%, 8 h 100%, cRD2D3: SIF 4 h 96%, 8 h 87.2%, SGF 4 h 99.5% and 8 h 99.7% Figure 3a,b). Additionally, we tested the stability of both d-peptides in human plasma and human liver microsomes. In plasma, both d-peptides were stable up to approximately 90% after 48 h (RD2D3: 8 h 84.7%, 24 h 91.9% and 48 h 93.7% and cRD2D3: 8 h 91.8%, 24 h 93.7% and 48 h 92.4%, Figure 3c). In liver microsomes, both d-peptides were slightly metabolized to approximately 25% (RD2D3: 8 h 81.9% and 24 h 78.2% and cRD2D3: 8 h 89.2% and 24 h 73.6%, Figure 3d).

### 2.2. Tg-SwDI Mice Develop Cognitive Deficits at 12 Months of Age Compared to WT Mice

**Figure 4:**
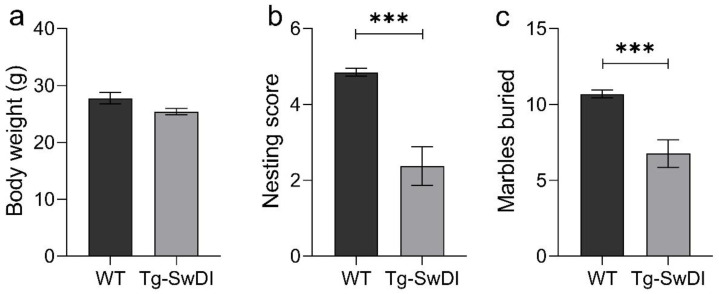
Basic phenotypic analysis of Tg-SwDI mice. No difference in body weight between Wild type (WT) and Tg-SwDI mice (**a**), but significant differences in nesting behavior (**b**) and marble burying (**c**) were found. Tg-SwDI mice formed a less mature nest and buried less marbles. Data are represented as mean ± SEM, all unpaired two-tailed *t* test, *** *p* < 0.001 (WT *N* = 13 and Tg-SwDI *N* = 12).

In all conducted experiments (nesting behavior, marble burying, open field test and Morris water maze (MWM)) Tg-SwDI mice showed an altered behavior compared to WT mice, but there was no significant difference in the body weight of WT compared to Tg-SwDI mice (Figure 4a). Analyses of some basic behavioral characteristics of Tg-SwDI resulted in phenotypic alterations. Compared to WT mice, Tg-SwDI mice formed a less mature nest (unpaired two-tailed *t* test, *p* < 0.001, Figure 4b). Furthermore, they displayed impaired digging behavior in direct comparison to WT mice of the same age, indicating a less inquisitive behavior (unpaired two-tailed *t* test, *p* < 0.001, Figure 4c).

**Figure 5:**
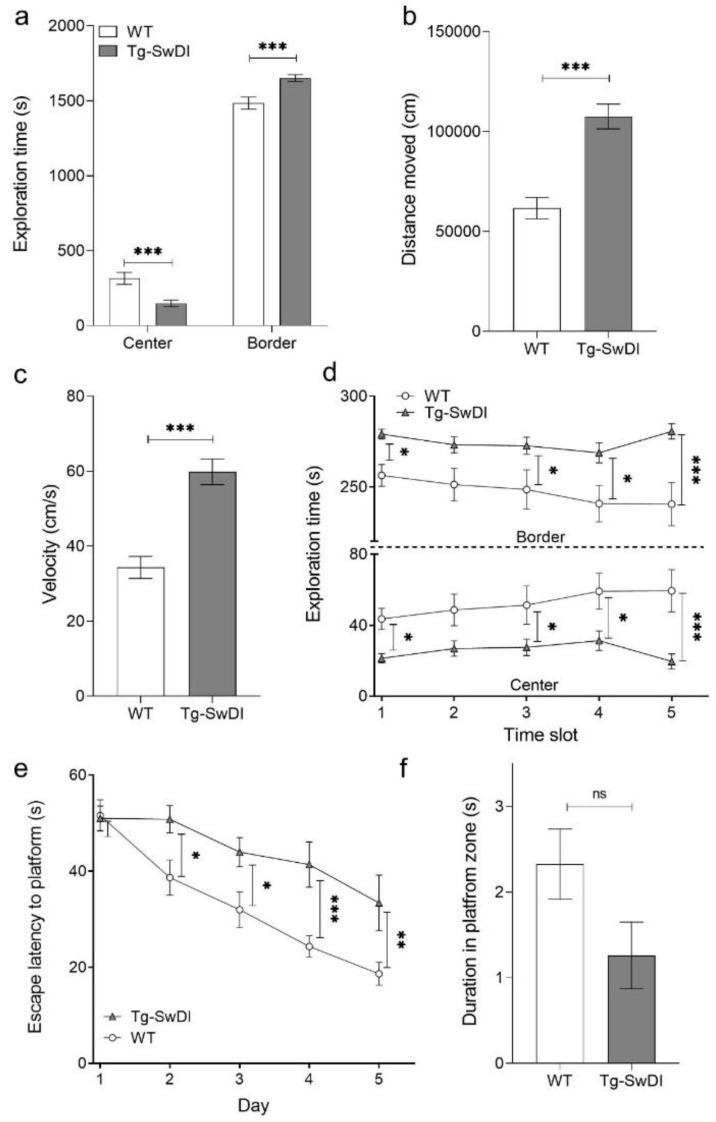
Tg-SwDI mice developed phenotypic deficits in the open field test and showed cognitive spatial impairment in the Morris water maze. In order to analyze some general aspects of the behavior of Tg-SwDI compared to wild type (WT) mice, an open field test was conducted, where mice were allowed to explore a square shaped arena freely for 30 min. The analysis of the test revealed that Tg-SwDI mice explored less the center of the arena (**a**) (two-way ANOVA genotypes *p* < 0.0001, Fisher LSD post hoc analysis WT vs. Tg-SwDI center and border *p* < 0.0001). Furthermore, they showed a reduced covered distance (**b**) and a reduced velocity (**c**) (both two-tailed *t*-test *p* = 0.0001). By analyzing the habituation effect of WT and Tg-SwDI mice to the arena, it was obvious that transgenic mice explored the center of the arena less than WT mice, indicating a reduced habituation effect to the arena (**d**) (two-way RM ANOVA, genotype *p* = 0.002, Fisher LSD post hoc analysis, WT vs. Tg-SwDI slot one *p* = 0.047, slot two n.s. (*p* = 0.052), slot three *p* = 0.034, slot four *p* < 0.014 and slot five *p* < 0.001). In the Morris water maze, a significant difference was detectable in the performance during the training of WT and Tg-SwDI mice starting at day 2, indicating spatial memory deficits (**e**) (two-way RM ANOVA, genotype *p* = 0.001, Fisher LSD post hoc analysis, WT vs. Tg-SwDI day one n.s. (*p* = 0.91), day two *p* = 0.018, day three *p* = 0.091, day four *p* < 0.001 and day five *p* = 0.004)). During the probe trial, WT mice spent more time in the platform zone than Tg-SwDI mice, indicating impairments with memory retrieval (**f**). Data are shown as mean ± SEM, * *p* < 0.05, ** *p* < 0.01 and *** *p* < 0.001 (WT *N* = 13 and Tg-SwDI *N* = 12).

While performing the open field test, a significant difference was found between the time WT and Tg-SwDI spent in the center and border zone (two-way ANOVA genotypes *p* < 0.0001, Fisher LSD post hoc analysis WT vs. Tg-SwDI center and border *p* < 0.0001, Figure 5a). By analyzing the travelled duration, Tg-SwDI covered an almost two-times longer distance than WT mice (two-tailed t-test, *p* < 0.0001, Figure 5b). Furthermore, Tg-SwDI mice were moving much faster than WT mice (two-tailed t-test, *p* < 0.0001, Figure 5c). Moreover, by analysis of single time slots (each slot 5 min) WT mice showed a clear habituation effect to the arena (Figure 5d). At the beginning of the test period, the center had an aversive effect to the mice, since they tended to avoid open areas. After a while, the WT mice explored the center of the arena more and more. In contrast, Tg-SwDI mice do not show this habituation effect (two-way RM ANOVA, genotype *p* = 0.002, Fisher LSD post hoc analysis, WT vs. Tg-SwDI slot one *p* = 0.047, slot two n.s. (*p* = 0.052), slot three *p* = 0.034, slot four *p* < 0.014 and slot five *p* < 0.001).

In order to analyze spatial memory, we conducted an MWM. On the first day of training, WT and Tg-SwDI mice performed almost equally (Figure 5e,f). Starting at day two, WT mice found the hidden platform significant faster than Tg-SwDI mice (two-way RM ANOVA, genotype *p* = 0.001, Fisher LSD post hoc analysis, WT vs. Tg-SwDI day one n.s. (*p* = 0.91), day two *p* = 0.018, day three *p* = 0.091, day four *p* < 0.001 and day five *p* = 0.004, Figure 5e). A slight learning effect could be seen for both WT and Tg-SwDI mice. However, this effect was much more pronounced in the WT mice. During the probe trial, WT mice spent more time in the platform zone than Tg-SwDI mice, indicating an improved memory retrieval (two-tailed t-test, *p* = 0.073, Figure 5f). Concluding, Tg-SwDI mice developed distinct cognitive deficits as detected by the MWM with 12 months of age.

### 2.3. RD2D3 Showed Superior Efficacy to Improve Phenotypic Deficits over cRD2D3

**Figure 6:**
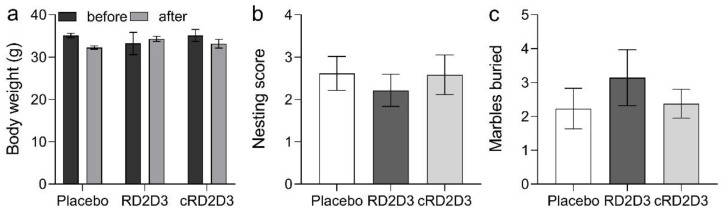
General behavior of RD2D3 and cRD2D3 treated tg-SwDI mice compared to placebo. There were no significant differences in all analyzed groups, neither in the body weight (**a**) nor in the nesting behavior (**b**) or marble burying (**c**). Data are represented as mean ± SEM (placebo *N* = 13, RD2D3 *N* = 14 and cRD2D3 *N* = 12).

All mice, regardless of treatment, showed no changes in their general appearance. Compared to d-peptide treated mice, placebo treated mice displayed a decrease in body weight (before vs. after treatment: placebo 35.1 ± 0.5 g vs. 32.3 ± 0.4 g, RD2D3 33.2 ± 2.6 g vs. 34.3 ± 0.6 g and cRD2D3 35.1 ± 1.4 g vs. 33.2 ± 1.0 g), but not to a significant extent (Figure 6a). There was no difference in the behavior of RD2D3 or cRD2D3 treated mice compared to placebo treated mice, neither in the nesting behavior, nor in the marble burying test. (Figure 6b,c).

**Figure 7:**
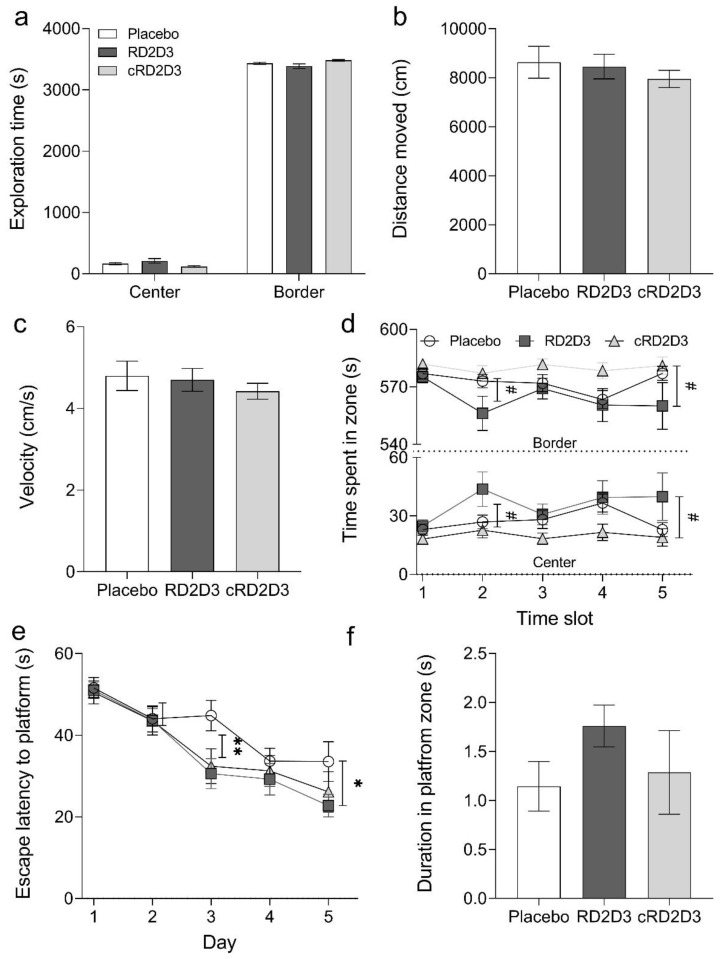
Treatment with RD2D3 significantly improved the phenotype of Tg-SwDI mice compared to placebo and cRD2D3 treated mice. An open field test was conducted to analyze and compare the exploratory and anxiety related behavior of RD2D3 and cRD2D3 treated Tg-SwDI mice compared to placebo treated mice (**a**–**d**). Mice were allowed to freely explore a square shaped arena for 30 min (imaginarily divided into center and border zone (**a**)). While there was no difference in the exploratory behavior or in the traveled distance or velocity, there was a significant habituation effect of RD2D3 treated mice to the arena (**d**). This effect was completely absent in cRD2D3 treated mice (**d**). Additionally, a Morris water maze (MWM) was performed in which mice were trained for 5 days to find a hidden platform (**e**). Both treatment groups (RD2D3 and cRD2D3) found the hidden platform faster than placebo treated mice and spent more time in the platform zone during the probe trial (**f**). Data are shown as mean ± SEM (placebo *N* = 13, RD2D3 *N* = 14 and cRD2D3 *N* = 12). ** *p* < 0.01 and * *p* < 0.05.

During the total open field test duration, no difference between treated or non-treated Tg-SwDI was detectable (Figure 7). Neither in the time the treatment groups spent in the center or border zone (Figure 7a) nor in the covered distance or in the velocity (Figure 7b,c). Taken a closer look, it was also possible to analyze the habituation effect of all mice to the arena. RD2D3 treated mice showed a slight but significant habituation effect to the arena compared to placebo treated mice (two-way RM ANOVA, *p* = 0.056, Fisher LSD post hoc analysis, placebo vs. RD2D3 slot 1 n.s., slot 2 *p* = 0.045, slot 3 n.s., slot 4 n.s. and slot 5 *p* = 0.44), this effect was completely absent in cRD2D3 treated mice. During each analyzed time slot, they spent almost the same time in the center or border, respectively (Figure 7d).

In this study, a MWM was performed to investigate, whether a treatment with RD2D3 or cRD2D3 improves the spatial memory or cognitive abilities of Tg-SwDI mice compared to placebo treated mice. As can be seen from Figure 7e, both compounds were able to improve the performance of Tg-SwDI mice in the MWM during the training phase. For RD2D3, this effect was statistically significant compared to placebo treated mice (two-way RM ANOVA treatment *p* = 0.11, Fisher post-hoc analysis placebo vs. RD2D3 day 1 n.s., day 2 n.n, day 3 *p* = 0.004, day 4 n.s. and day 5 *p* = 0.029). In order to test memory retrieval, a probe trial was conducted. Although not to a significant extent, there was a preference of RD2D3 treated mice spending more time in the platform zone (Figure 7f).

**Figure 8:**
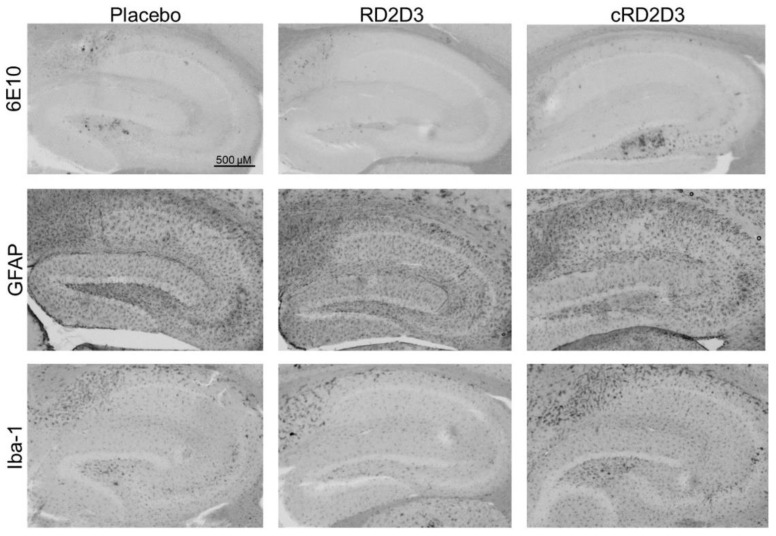
Immunohistochemical analysis of Aβ deposits and neuroinflammation. Representative images of hippocampus of placebo, RD2D3 and cRD2D3 treated mice (placebo *N* = 13, RD2D3 *N* = 14 and cRD2D3 *N* = 12).

In order to investigate, whether treatment with RD2D3 or cRD2D3 change the pathological characteristics of Tg-SwDI mice, several histological analyses were conducted. As demonstrated in Table 1 and Figure 8, neither RD2D3 nor cRD2D3 were able to reduce the amount of Aβ deposits as shown by 6E10 staining, nor reduce the number of activated astrocytes or microglia, as shown by GFAP or Iba-1 staining, respectively.

## 3. Discussion

More than 100 years have passed since AD was first described by Alois Alzheimer [19]. Meanwhile, many efforts have been made to find a curative or even disease modifying treatment for this devastating neurodegenerative disease [20]. One of the major pathological hallmarks of the disease are deposits of the so called Aβ-peptide. No toxic properties are attributed to Aβ in its monomeric, native form. Only a soluble intermediate product, the so-called Aβ-oligomers, are considered to be the disease-causing agent [3,18,21]. Our group has focused on the development of compounds, which directly destroy toxic Aβ-oligomers. These therapeutic substances are so-called d-peptides, consisting of 12–24 d-enantiomeric amino acid residues. The most promising compound so far is RD2. In the recent years, RD2 has been studied extensively [10,11,12,13]. The efficacy of this compound has been demonstrated in several studies in different mouse models, so RD2 is now in the process of proving its efficacy in humans in clinical trials [9]. Out of several drug optimization approaches, the linear tandem-d-peptide RD2D3 and its cyclic equivalent, cRD2D3, came forth [12,14,15,22]. Tandemization and cyclization of the developed d-enantiomeric peptides were postulated to increase the binding affinity to Aβ(1-42), to increase the efficacy to eliminate toxic Aβ-oligomers and to increase BBB penetration. This last point in particular was demonstrated by Schartmann et al. [22]. After the pharmacokinetic and some in vitro analyses of the d-peptides were conducted recently, we here performed an in-depth comparison of the in vitro potency of both d-peptides on wild type Aβ(1-42) and on the D/I Aβ mutation and a direct comparison of the d-peptide’s in vivo efficacy in Tg-SwDI mice.

During the last years, we could demonstrate that our developed d-peptides bound (mostly) to Aβ(1-42) in the micromolar range. This was also already proven for RD2D3 [14]. However, it was never analyzed if these compounds also bind to different Aβ-variants, e.g., the D/I mutation, although there have been several treatment studies conducted with a mouse model harboring this and other mutations [14,16,18,23]. In this study, we analyzed the binding affinity of both RD2D3 and cRD2D3 to Aβ(1-42) for a direct comparison, and the binding affinity of the abovementioned d-peptides to D/I Aβ. The results showed that both d-peptides exhibit similar binding affinities to Aβ(1-42) and D/I Aβ. However, this binding is at least twice as strong to D/I Aβ in comparison to Aβ(1-42).

Taking a closer look into the in vitro functionality of both d-peptides, we performed a ThT and a QIAD assay. Both d-peptides are capable to efficiently inhibit the Aβ(1-42) fibril formation at equimolar concentrations. One might speculate that RD2D3 was slightly more efficient since it also shifted the lag phase of the initial aggregation of Aβ(1-42). By analyzing the ability of both d-peptides to inhibit the D/I Aβ fibril formation, we could not detect a meaningful difference between the potency of both d-peptides. RD2D3 and cRD2D3 inhibit the D/I Aβ fibril formation at equimolar concentrations. Despite the higher binding affinity to D/I Aβ of both d-peptides compared to Aβ(1-42), both d-peptides show a superior potency to inhibit the Aβ(1-42)- than the D/I Aβ-fibril formation. Aβ-oligomer elimination efficacy was already proven for RD2D3 on Aβ(1-42) [12]. Here, we performed an additional QIAD assay to directly compare the Aβ-oligomer elimination efficacy of RD2D3 with cRD2D3. We were able to confirm that RD2D3 and cRD2D3 eliminate Aβ(1-42)-oligomers in vitro especially effective to about 90% or 80%, respectively. For the first time, we also conducted a QIAD assay with an Aβ mutation—D/I Aβ. We could demonstrate that both d-peptides can eliminate (more) efficiently D/I Aβ-oligomers in vitro to about 95% or 100%, respectively.

In order to verify the already demonstrated proteolytic stability of RD2D3 and cRD2D3 with a more sensitive method (HPLC analysis instead of thin layer chromatography with ^3^H-labeled d-peptide) and to extend the analysis, proteolytic stability tests in different (simulated) human body fluids were done. Analysis of the proteolytic stability of ^3^H-RD2D3 [15] and ^3^H-cRD2D3 [22] in human liver microsomes revealed that both d-peptides appeared to be completely stable. In contrast, the d-peptides were metabolized up to 25% after 8 h when analyzed by HPLC. This discrepancy can probably be explained by the fact that analysis by HPLC is many times more sensitive and accurate than analysis by thin-layer chromatography.

In the aforementioned study [22] it was described that cRD2D3 has an extraordinary superior pharmacokinetic profile in C57BL/6 mice compared to its linear equivalent RD2D3 after i.v. and i.p. administration [15,22]. One of the most outstanding properties of the cyclic d-peptide is its enormously long terminal half-life in plasma of more than 2 days (58 h). In comparison, the terminal half-life in plasma of RD2D3 was 2.3 h. Moreover, it was shown that cRD2D3 reached its site of action, the brain as a target organ, with concentrations up to four to five times higher than RD2D3.

After analyzing the in vitro profile of the compounds, we wanted to investigate whether the superior pharmacokinetic profile of cRD2D3 was also reflected in improved efficacy in the Tg-SwDI mouse model. For this purpose, we first performed a small in-house characterization of the mouse model. This served the purpose of confirming that the Tg-SwDI mice also develop the expected (cognitive) deficits in our hands and in our experimental setup. The implemented behavioral experiments (nesting behavior, marble burying, open field test and Morris water maze) gave proof that the Tg-SwDI mouse model develops general and cognitive phenotypic deficits in our hands at 12 months of age. Although development of pathology and especially of cognition impairment is often dependent on breeding cohorts and lab environments, our results are in good accordance with those reported previously [24,25,26,27]. After completion of the characterization study, we performed an i.p. treatment study with RD2D3 and cRD2D3 compared to placebo treated mice by use of Alzet osmotic minipumps. Treatment started with 11 months of age and mice were sacrificed after the pump duration of 28 days (at 12 months of age). The treatment start was based on the results of the characterization study, where cognition deficits were detected at 12 months of age. One might speculate that a treatment with an earlier age and a prolonged treatment duration might have also been sufficient. During the last days of treatment, several behavioral tests were conducted. Those tests indicated that RD2D3 had a more pronounced effect on the phenotypic deficits of Tg-SwDI mice than cRD2D3. The analysis of the open field test revealed a clear habituation effect of RD2D3 treated mice to the arena, comparable with the behavior of non-transgenic mice. In contrast, this effect was completely absent in cRD2D3 treated mice. The performed MWM suggested that both RD2D3 and cRD2D3 treatment improved the cognitive performance of Tg-SwDI mice compared to placebo treated mice. However, this effect reached significance only in the RD2D3 treated Tg-SwDI mice. The use of a larger number of mice would probably have resulted in this effect also being significant in the cRD2D3 treated Tg-SwDI mice. Histological analysis of the brains of all mice revealed no difference between the treatment groups, neither on Aβ-deposits nor on activated astrocytes or microglia. One might speculate that a administering a higher dose of the d-peptide concentration or a longer treatment duration would have resulted in a reduction of AD-associated pathology. Previous pharmacokinetic analyses of RD2D3 and cRD2D3 have shown that both d-peptides do reach the brain [6,7]. Summarized, RD2D3 appears to have a superior efficacy to ameliorate the phenotype of Tg-SwDI than cRD2D3 compared to placebo treated mice.

Referring to the hypothesis that cyclized d-peptides also show improved in vivo efficacy compared to linear d-peptides due to a more favorable pharmacokinetic profile [22] could not be confirmed in this study. In all tests performed, cRD2D3 treated Tg-SwDI mice showed similar or slightly worse behavior than RD2D3 treated mice. In conclusion, the superior pharmacokinetic profile of cRD2D3 was not sufficient to translate into improved in vivo efficacies.

## 4. Materials and Methods

### 4.1. Peptides

The d-peptides RD2D3 (ptlhthnrrrrrrprtrlhthrnr) and cRD2D3 (ptlhthnrrrrrrprtrlhthrnr) with amidated C-terminus were purchased as lyophilized powder with >95% purity from peptides&elephants (Henningsdorf, Germany) and Cambridge peptides (UK), respectively.

Synthetic Aβ(1-42) and (Gln^22^, Asn^23^)-Amyloid β-Protein (1-40) (D/I Aβ) with >95% purity were purchased as lyophilized powder from Bachem (Bubendorf, Switzerland). Lyophilized Aβ-species were dissolved overnight in HFIP (1,1,1,3,3,3-hexafluoro-2-propanol, Sigma-Aldrich, Darmstadt, Germany). Aliquots were stored at −20 °C until further processing. Before usage, Aβ was lyophilized and dissolved in 10 mM sodium phosphate buffer, pH 7.4.

### 4.2. In Vitro Potency

#### 4.2.1. Binding Affinity

The dissociation constant (K_D_) of RD2D3 and cRD2D3 binding to Aβ(1-42) and D/I Aβ was determined by SPR spectroscopy using a Biacore T200 instrument (Biacore, GE Healthcare, Uppsala, Sweden). Aβ(1-42) or D/I Aβ was used as the ligand, while RD2D3 and cRD2D3 were used as the analyte or vice versa.

By using Aβ(1-42) or D/I Aβ as a ligand, it was immobilized onto a series S CM-5 sensor chip (GE Healthcare, Uppsala, Sweden) by amine coupling. In short, the flow cells were activated by a mixture of 50 mM N-hydroxysuccinimide (NHS) and 16.1 mM N-Ethyl-N’-(dimethylaminopropyl)carbodiimide (EDC) (XanTec, Düsseldorf, Germany) for 7 min. Aβ(1-42) or D/I Aβ were dissolved to a final concentration of 50 µg/mL in 10 mM sodium acetate pH 5 (AppliChem, Darmstadt, Germany) and injected over one of the activated flow cells to a final signal of 1700 RU. After the immobilization, the ligand and reference flow cells were quenched by injecting 1 M ethanolamine pH 8.5 (XanTec, Düsseldorf, Germany) for 7 min. For the determination of the K_D_, multicycle kinetic experiments were performed with 10 mM HEPES + 50 mM NaCl (AppliChem, Darmstadt, Germany) pH 7.4 as the running buffer at 25 °C and at a flow rate of 30 µL/min. The peptides were diluted in the running buffer to the following concentrations: 20 µM, 10 µM, 5 µM, 2.5 µM, 1.25 µM and 0.625 µM. All samples were injected over the flow cells for 180 s, followed by a dissociation step of 600 s with running buffer. Regeneration of the sensor chip was accomplished by a 45 s injection of 2 M of guanidinium hydrochloride (AppliChem, Darmstadt, Germany). The reference flow cell and buffer injections (c = 0 nM) were used for double referencing of the sensorgrams. For data evaluation, the sensorgrams were fitted by the steady-state affinity model implemented in the Biacore T200 Evaluation Software 3.2.

By using the compounds (RD2D3 and cRD2D3) as ligands, they were immobilized on two separate channels on a series S CM-5 sensor chip (Cytiva, GE Healthcare, Uppsala, Sweden) by amine coupling. Both flow cells on each channel were activated with a mixture of 50 mM N-hydroxysuccinimide (NHS) and 16.1 mM N-ethyl-N’-(dimethylaminopropyl)carbodiimide (EDC) (XanTec, Düsseldorf, Germany) for 7 min. The peptides were diluted to 50 µg/mL in 10 mM maleic acid pH 6 (AppliChem, Darmstadt, Germany) and injected over flow cell two of each channel to a final signal between 600 and 900 RU. After the peptides were immobilized, the ligand and reference flow cells of each channel were quenched by injecting 1 M ethanolamine pH 8.5 (XanTec, Düsseldorf, Germany) for 7 min. For the determination of the K_D_ multicycle kinetic experiments were performed with 10 mM HEPES + 50 mM NaCl (AppliChem, Darmstadt, Germany) pH 7.4 as the running buffer at 25 °C and at a flow rate of 30 µL/min. Aβ(1-42) or D/I Aβ were diluted in the running buffer to the following concentrations: 10 µM, 3.3 µM, 1.1 µM, 0.37 µM, 0.12 µM, 0.045 µM, 0.014 µM and 0.0045 µM. All samples were injected over the flow cells for 180 s, followed by a dissociation step of 600 s with running buffer. Regeneration of the sensor chip was accomplished by a 45 s injection of 2 M guanidinium hydrochloride (AppliChem, Darmstadt, Germany). The reference flow cell of each channel and the buffer injections (c = 0 nM) were used for double referencing of the sensorgrams. For data evaluation, the sensorgrams were fitted by the steady-state affinity model implemented in the Biacore Insight Evaluation Software version 3.0.

#### 4.2.2. Thioflavin-T Assay

Using the ThT assay, the potency of RD2D3 and cRD2D3 to inhibit the fibril formation of Aβ(1-42) and D/I Aβ was analyzed. For this purpose, 10 µM Aβ(1-42) or D/I Aβ were incubated with 10 µM of the corresponding d-peptide and 5 µM ThT. ThT fluorescence was monitored over 24 h every 6 min at λex = 440 nm and λem = 490 nm in a fluorescence plate reader (Clariostar, BMG Labtech, Ortenberg, Germany) at RT. Correction was done using all supplements without Aβ and d-peptide (blank).

#### 4.2.3. QIAD Assay

According to Brener et al., a QIAD assay was performed in order to evaluate the Aβ(1-42) or D/I Aβ oligomer elimination efficacies of RD2D3 and cRD2D3 [18]. In short, 80 µM lyophilized Aβ(1-42) or 40 µM D/I Aβ was preincubated for 2 h or 15 min, respectively, to enrich Aβ-oligomers. Afterwards, either 20 µM RD2D3 or cRD2D3 for Aβ(1-42) or 10 µM RD2D3 or cRD2D3 for D/I Aβ were added to the preincubated solution and coincubated for additional 30 min. Subsequently, the samples were loaded on the top of a density gradient (5–50% (*w*/*v*) iodixanol (OptiPrep, Sigma-Aldrich, Darmstadt, Germany)) followed by an ultracentrifugation step (3 h at 4 °C and 259000× *g* (Optima TL-100, Beckman Coulter, Brea, CA, USA)). Following the ultracentrifugation, 14 fractions (140 µL each) were harvested by upward displacement. Fraction 15 contains the dissolved pellet in 6 M guanidine hydrochloride solution. Top fractions 1–2 contained Aβ-monomers, fractions 4–6 contained the Aβ-oligomers, which are of special interest, and the bottom fractions 11–14 contained high molecular weight (co-)precipitates or aggregated Aβ. Aβ(1-42) or D/I Aβ concentrations of each fraction were determined via analytical RP-HPLC and UV absorbance detection at 214 nm.

#### 4.2.4. Proteolytic Stability

Both compounds have already been extensively studied with respect to their in vivo pharmacokinetic profiles [15,22]. Here, we performed some additional test to investigate the proteolytic stability of the compounds in simulated gastric (SGF) and intestinal fluid (SIF) and human plasma and human liver microsomes. The experiments were performed as described previously [28]. According to the guidelines of the European Pharmacopoeia 7.0, SGF and SIF were prepared. Plasma was purchased from Biotrend with K3-EDTA as an anticoagulant (Biotrend, Köln, Germany). Human liver microsomes were pooled from different donors and purchased from Sekisui XenoTech (Kansas City, KS, USA).

In order to determine whether the compounds RD2D3 and cRD2D3 are stable or would be metabolized, 150 µM of each compound was incubated in triplicate for defined time points slightly shaking at 37 °C in the media described above (SGF and SIF: 4 and 8 h, plasma: 8, 24 and 48 h and microsomes: 8 and 24 h). Incubation was stopped by precipitating the proteins. For this, 3% trichloroacetic acid (*w*/*v*) was added to the respective sample under vortexing followed by a centrifugation step (14,000× *g* at 4 °C for 5 min). For analysis, supernatants containing the compounds were collected. The precipitated media without compound was used as a control and as reference for the quantification served each medium with compound where the reaction was stopped immediately by precipitating the proteins. All samples were analyzed by RP-HPLC as previously described [28].

### 4.3. In Vivo Efficacy

#### 4.3.1. Animals

In this study, the Tg-SwDI AD mouse model was used. Tg-SwDI were first described by Davis et al. in 2004 and carried the human APP gene (isoform 770) with the Swedish (K670N/M671L), Dutch (E693Q) and Iowa (D694N) mutations under the control of the mouse Thy1 promoter [24]. Aβ-depositions can be found starting with three months of age and cognition deficits can be detected as early as three months of age in the Barnes maze [24,26]. Tg-SwDI mice are known to show distinct AD-associated pathology. This includes Aβ-deposition in various areas of the brain (particularly in the cortex and hippocampus) and associated gliosis. Gliosis is characterized by the presence of activated microglia and astrocytes, especially in those regions where Aβ-deposits are also found. In addition to Aβ-deposits in the tissue, this mouse model is particularly characterized by a so-called cerebral amyloid angiopathy (CAA). This means that Tg-SwDI mice show increased Aβ-deposits in cerebral vessels.

Tg-SwDI mice were ordered by the Jackson Laboratory (C57BL/6-Tg(Thy1-APPSwDutIowa)BWevn/Mmjax, Jackson Laboratory, Bar Harbor, ME, USA) and bred in-house in a controlled environment on a light/dark cycle (12/12 h), with 54% humidity and a temperature of 22 °C. Food and water were available ad libitum.

#### 4.3.2. Ethical Approval

All animal experiments were done in accordance with the German Law on the protection of animals and approved by the Landesamt für Natur, Umwelt und Verbraucherschutz (LANUV) North-Rhine-Westphalia, Germany (AZ84-02.04.2016.A523).

#### 4.3.3. Characterization

In order to perform a short in-house characterization of the Tg-SwDI mouse model, we used 12 months old female homozygous (*n* = 12) and corresponding wild type (*n* = 13) mice to verify cognition deficits at the same age at which the treatment study should be performed. Mice were tested in several behavioral tests to identify differences between Tg-SwDI and WT mice.

#### 4.3.4. Nesting Behavior

Building a nest is a core behavior of mice, not just for maternal nesting. It is crucial for mice for shelter from the environment and protection against predators. Here, a protocol after Deacon was used. In brief, 1 h before the dark phase of the animal house, mice were single placed in a new cage with a fresh nestlet (Sniff, Soest, Germany). The next morning, built nests were scored from 1 to 5, whereby 1 represents no nest and 5 represents a perfect nest [29].

#### 4.3.5. Marble Burying

Digging and burrowing is a fundamental behavior of mice to find and hide food and to build a nest. Several drugs or genetic modifications may alter the digging behavior of mice. For analysis of digging behavior, the marble burying test was performed after Deacon. Mice were single placed in a new cage with approximately 5 cm deep wood chip bedding. On the bedding, 12 glass marbles (diameter: 1.6 cm and weight: 5.3 g) were laid down in a predefined pattern. After 30 min, the number of marbles buried was counted [30].

#### 4.3.6. Open Field Test

Analysis of explorative and anxiety related behavior of mice can be done by use of the so-called open field test [31]. Following a 30 min habituation phase in the experiment’s room, the mice were placed in a square-shaped arena (45 cm × 45 cm × 45 cm) for an additional 30 min, imaginarily divided into two zones: center and border zone (center: 19 cm × 19 cm and border: space around the center zone). During the 30 min exploration, mice were recorded with a camera driven tracking system (Ethovision 15, Noldus, Wageningen, The Netherlands). For analysis, the duration the mice stayed in each zone was evaluated. Moreover, several time slots (1: 0–5 min, 2: 5–10 min, 3: 10–15 min, 4: 15–20 min and 5: 20–25 min) were analyzed independently to determine habituation behavior of the mice.

#### 4.3.7. Morris Water Maze

Investigation of spatial learning can be conducted by the use of the MWM. The MWM is one of the most widely used tests to investigate cognitive impairments, especially spatial memory, in neuroscience [32]. The water maze we have used consists of a circular white pool (120 cm in diameter and 60 cm in height). Rendering of the water was ensured by adding a non-toxic white coloring solution. To conduct the test, the pool is imaginarily subdivided into four quadrants (north-east, south-east, south-west and north-west). In the middle of the target quadrant, an invisible round platform was placed 1 cm below the surface. The used protocol was modified after the original one from Morris et al. [33]. During the training period, mice were allowed to swim for 60 s or until they found the hidden platform. If they did not find the hidden platform, they were set on the platform for 10 s to orient themselves before they were returned to their cages. On each of the five training days, the mice had to swim four trials, each trial starting from a different quadrant, with the order changing each day. To avoid a decrease of body temperature of the mice, they were placed under a heating lamp for 60 s between each trial. On the sixth day, the mice had to swim freely without a hidden platform (probe trial). During each trial, the mice were recorded with a video driven tracking system (Ethovision 15, Noldus, Wageningen, The Netherlands). The following parameters were analyzed: escape latency to find the hidden platform during the training phase or duration in platform zone during the probe trial.

### 4.4. Treatment

Eleven months old Tg-SwDI were treated for 28 d i.p. by use of an osmotic minipump (Alzet osmotic pumps, Modell 1004, Charles River, Wilmington, MS, USA) with a daily dosage of 8 mg/kg peptide (RD2D3 (*n* = 14) or cRD2D3 (*n* = 12)) in PBS (pH 7.4) or vehicle (Placebo (*n* = 13), PBS, pH 7.4). The minipumps and the i.p. application route have been used to achieve continuous application of the study drug. After surgery, mice were monitored daily the following three days and twice each week until the end of the treatment. A loss of body weight and severe conspicuities were defined as exclusion criteria. No mouse was affected by these exclusion criteria. During the last one and a half weeks of treatment, different behavioral tests (nesting behavior, marble burying, open field test and MWM) were conducted with all mice.

Since RD2D3 did not show any efficacy in vivo after oral administration [12], but after i.p. administration [14], we decided to perform an i.p. treatment study for the direct comparison of the linear and cyclic d-peptide.

### 4.5. Histology

After the treatment duration was finished, the mice were anesthetized (intraperitoneal injection of 100 mg/kg ketamine (bela-pharm, Vechta, Germany) and 0.3 mg/kg medetomidine (Dormilan, alfavet, Neumünster, Germany) and transcardially perfused with ice-cold PBS. Subsequently, brains were removed and one hemisphere was frozen. The other hemisphere was fixed overnight in 4% paraformaldehyde, followed by a post-fixation and cryoprotection in 30% sucrose for an additional day. For histological analysis, 40 µm free floating sections were prepared on a cryostat. In total, eight series of six sections each were cut sagittally through the brain. One series of each brain was used for the following stainings: 6E10 (Biolegend, San Diego, CA, USA) for Aβ-deposits, GFAP (Agilent, Santa Clara, CA, USA) for reactive astrocytes and Iba-1 (Fujifilm, Neuss, Germany) for activated microglia. In brief, sections were washed in TBST (TBS with 1% Triton X-100), followed by an overnight incubation with the primary antibody (6E10 1 µg/mL, GFAP 1 µg/mL and Iba-1 0.5 µg/mL). The very next day, sections were washed in TBST, followed by the incubation with the secondary antibody (6E10 goat anti-Mouse IgG (H + L) secondary antibody 1.3 µg/mL, GFAP and Iba-1 goat anti-rabbit IgG (H + L) secondary antibody, 1.5 µg/mL, all Thermo Fisher, Germany) for 2 h at RT. After an additional washing step in TBST, stainings were visualized with the use of 3,3′ diaminobenzidine (DAB) enhanced with saturated nickel ammonium sulphate solution. Afterwards, sections were mounted with DPX Mountant (Sigma-Aldrich, Darmstadt, Germany).

To avoid any irregularities in the staining results, all stainings were performed in one batch and in one microscopy session. Zeiss SteREO Lumar V12 microscope and the according software (Zeiss AxioVision 6.4 RE) was used for visualization. Quantification was done with ImageJ (NIH, USA) and CellProfiler (Broad Institute, Cambridge, MA, USA).

### 4.6. Statistics

All statistical analyses were performed using GraphPad Prism 8 (GraphPad Software, Inc., San Diego, CA, USA) or SigmaPlot Version 11 (Systat Software, Düsseldorf, Germany). In vitro data are represented as mean ± SD and in vivo data as mean ± SEM. Normal distributed data were analyzed by use of an unpaired one- or two-tailed *t*-test or one-way analysis of variance (ANOVA) with Tukey post hoc analysis. Not normal distributed data were analyzed by use of the Kruskal–Wallis test with Dunn’s multiple comparison test. The MWM and open field test were analyzed by a repeated measure ANOVA with Fisher or Bonferroni post hoc analysis. Data with *p* < 0.05 were stated as significant.

## 5. Conclusions

In this study, we analyzed the in vitro potency of two of our developed d-enantiomeric d-peptides on an artificial contribution of two familial mutations within the Aβ sequence, namely the Dutch and Iowa mutations, for the first time. The analysis of the in vitro profile revealed that RD2D3 and cRD2D3 have a similar potency on both analyzed Aβ-species, regarding the potency to inhibit the Aβ-fibril formation and the potency to eliminate toxic Aβ-oligomers. Referring to the hypothesis that a cyclization of our d-peptides might show superior in vivo efficacy could not be confirmed in this study. Compared to linear RD2D3, cyclic cRD2D3 failed to show superior efficacy.

## Figures and Tables

**Table 1:** Immunohistochemical investigations of RD2D3 and cRD2D3 treatment on Aβ deposits and neuroinflammation. Treatment with neither RD2D3, nor cRD2D3 did reveal any increase or decrease in Aβ deposits (6E10), activated astrocytes (GFAP) or microglia (Iba-1). IR: immunoreactivity. Data are represented as mean ± SEM. IR: immunoreactivity (placebo *N* = 13, RD2D3 *N* = 14 and cRD2D3 *N* = 12).

	6E10 IR (%)		GFAP IR (%)		Iba-1 IR (%)	
	Cortex	Hippocampus	Cortex	Hippocampus	Cortex	Hippocampus
Placebo	1.8 ± 0.7	2.5 ± 0.7	43.8 ± 2.0	39.8 ± 1.0	8.7 ± 1.7	11. 5 ± 1.1
RD2D3	1.0 ± 0.2	2.5 ± 0.5	40.6 ± 2.2	38.0 ± 1.2	8.1 ± 1.6	8.6 ± 1.3
cRD2D3	1.1 ± 0.2	4.2 ± 0.7	43.1 ± 1.8	39.5 ± 1.0	8.1 ± 1.6	10.2 ± 1.8

## Data Availability

The data presented in this study are available in this article.
